# Complex dissociation following maternal suicide attempt in a 17-year-old female: a case report

**DOI:** 10.1186/s13256-025-05673-6

**Published:** 2025-11-19

**Authors:** Shota Hanyu

**Affiliations:** 1 Department of Psychiatry, Aino Hanazono Hospital, 2-6-1 Hanazono, Ibaraki, Osaka 567-0017 Japan; 2 Department of Psychiatry, Medical Research Institute, Kitano Hospital, 2-4-20 Ōgimachi, Kita-Ku, Osaka-Shi, Osaka 530-8480 Japan

**Keywords:** Dissociative amnesia, Dissociative stupor, Conversion disorder, Psychological trauma, Case report

## Abstract

**Background:**

Dissociative disorders involve disruptions in memory, identity, sensory awareness, and motor control, often triggered by psychological distress. Dissociative amnesia may appear as either retrograde or anterograde memory loss, and dissociative stupor as well as conversion disorder are also considered part of the dissociative disorders spectrum. Cases presenting with generalized amnesia (both retrograde and anterograde), dissociative stupor, and conversion disorder are rare. Here, I report a unique case of a 17-year-old female exhibiting this combination of symptoms following her mother’s depressive relapse.

**Case presentation:**

A 17-year-old Japanese female developed complete amnesia 3 weeks before presentation. Her mother, diagnosed with depression, had been hospitalized. The patient had a history of exposure to domestic violence and had taken on a caregiving role for her mother. She initially experienced episodic stupor, involuntary eye deviation, and transient unconsciousness, followed by generalized amnesia affecting personal, semantic, and procedural memory. Neurological and medical evaluations ruled out organic causes, leading to a psychiatric referral. As amnesia improved, she recalled witnessing her mother’s suicide attempt 2 years prior. Her symptoms were diagnosed as dissociative amnesia, dissociative stupor, and conversion disorder, attributed to psychological distress. Supportive psychotherapy, psychoeducation, and environmental stabilization led to symptom resolution without medication.

**Conclusion:**

This case highlights a rare presentation of dissociative amnesia with stupor and conversion disorder. The diagnosis was initially challenging owing to amnesia masking the stressor. Careful exclusion of organic causes and thorough psychological assessment were key. This case underscores the importance of identifying underlying trauma in complex dissociative presentations.

## Background

Dissociative disorders involve impairments in the usual coordination of functions such as memory, sense of identity, sensory perception, and voluntary motor activity. These disorders are often triggered by traumatic events or severe psychological distress [1]. Dissociative amnesia, a key subtype within dissociative disorders, is typically divided into retrograde and anterograde forms. Conditions such as dissociative stupor and conversion disorder are also included within the dissociative spectrum [2].

Mixed presentations combining dissociative and conversion phenomena (that is, functional neurological symptom disorders) are permissible within major diagnostic frameworks, yet comprehensive descriptions—particularly in adolescents—remain limited. Such cases may present with fluctuating unresponsiveness, abnormal movements or postures, and amnesia in close temporal relation to psychological stressors, and they require careful differential diagnosis from epilepsy, catatonia, syncope, and other neurological conditions. In line with major diagnostic systems (ICD-10/ICD-11 and DSM-5), dissociative amnesia, dissociative stupor, and conversion disorder can co-occur in response to severe stress, and trauma-informed assessment is essential. However, cases presenting with generalized dissociative amnesia involving both retrograde and anterograde memory impairment, along with dissociative stupor and conversion disorder, are rare. Here, I report a unique case with this combination of dissociative symptoms.

This article is based on a case report first published in Japanese in Seishinka Chiryo Gaku (The Japanese Journal of Psychiatric Treatment), Volume 39, Issue 7, pages 805–808, in 2024 [3]. It is republished here in English as a secondary publication, adapted and approved by both the original publisher and the editorial board of the *Journal of Medical Case Reports*, in accordance with the International Committee of Medical Journal Editors (ICMJE) guidelines for secondary publication.

## Case presentation

To protect confidentiality, all dates are expressed relative to the initial psychiatric presentation, which is defined as *T* = 0 (initial presentation). Earlier or later events are described as negative or positive intervals from this time point. A relative timeline of key clinical events is summarized in Table 1. A 17-year-old Japanese female presented with complete amnesia that had begun 3 weeks prior. Her mother, diagnosed with depression 4 years earlier, had been hospitalized approximately 1 month before the initial psychiatric presentation (*T* =  −1 month) owing to worsening symptoms. Around age 13 years, the patient had been exposed to repeated conflict and domestic violence involving her mother’s partner. Although her mother did not express suicidal ideation at the time, marked mood fluctuations were evident. As the only daughter among four siblings, the patient had assumed an emotional caregiving role from an early age. She lived with her grandmother and younger brother, while her older siblings had already moved out. Despite a complex family background, her developmental milestones were normal, and she demonstrated strong academic and athletic performance, particularly in soccer and softball. Her medical history was unremarkable.

**Table 1:** Relative timeline of key clinical events

Time (relative to initial psychiatric presentation)	Event
*T* = −5 months	Found unconscious at school; minimal responsiveness, spontaneous recovery over 3 hours
*T* = −2 months	Collapsed in restroom with fine tremors and mild oxygen desaturation; recovered in transit
*T* = −1 month	Daily transient unresponsiveness began; mother hospitalized for depression
*T* = −3 weeks	Unresponsiveness ceased; onset of generalized retrograde amnesia
*T* = 0 (initial presentation)	Psychiatric referral owing to persistent amnesia
*T* = +12 days	Partial memory return; recollection of mother’s suicide attempt 2 years earlier

This table summarizes the key clinical events from the patient’s initial stupor episode to the recollection of her mother’s suicide attempt 2 years earlier. Dates are presented relative to the initial psychiatric presentation (*T* = 0) to protect confidentiality, consistent with journal guidance

At *T* =  −5 months, she was found unconscious in a school hallway, showing minimal responsiveness before gradually regaining consciousness over 3 hours. At *T* =  −2 months, she collapsed in a restroom, displaying fine tremors and mild oxygen desaturation, but recovered during transportation to the hospital. No structural, cardiopulmonary, or metabolic abnormalities were documented in the available records. The transient desaturation was considered most plausibly related to shallow breathing during reduced responsiveness. She also experienced a single prolonged episode of sustained upward eye deviation lasting several hours without documented recurrence. From approximately *T* =  −1 month, she had daily episodes of transient unresponsiveness of varying durations. These episodes resolved about 3 weeks before *T* = 0, and were followed by profound retrograde amnesia involving personal, semantic, and procedural memory. Intermittent anterograde amnesia was also noted, with difficulty retaining recent interactions.

Owing to the persistence of symptoms, the patient initially consulted an internal medicine department. Laboratory tests, chest X-ray, electrocardiogram, and brain magnetic resonance imaging (MRI) revealed no abnormalities. She was subsequently referred to psychiatry at *T* = 0 (initial presentation) under suspicion of a psychogenic etiology. Records from prior emergency evaluations were unavailable, and the patient had no recollection of those events. During the psychiatric assessment, she was accompanied by her grandmother and presented as calm, socially appropriate, and cooperative. Although she reported difficulty recognizing familiar faces, names, and locations, she displayed no distress, and often smiled and spoke positively about school and friendships despite the ongoing memory impairment.

The initial working diagnoses considered were epileptic seizures accompanied by amnesia, dissociative amnesia, and dissociative stupor. Electroencephalogram (EEG) and neurological examinations conducted by neurology ruled out epilepsy. She initially denied significant past psychological trauma, making dissociative disorders less likely at first. However, at *T* =  +12 days (early follow-up after the initial visit), her amnesia had been improving, and she recalled witnessing her mother’s suicide attempt by hanging 2 years prior. She had experienced distressing flashbacks at the time but had not disclosed them, as her academic and daily activities had been unaffected. Her mother’s recent worsening depression was identified as another significant stressor.

Considering her history, generalized amnesia involving significant life events was attributed to intense psychological stress from her mother’s depression and previous suicide attempt, substantially impairing social functioning. With no evidence of substance use, neurological conditions, medical disorders, excessive fatigue, or other psychiatric disorders, dissociative amnesia was diagnosed. Prior to amnesia onset, she had experienced episodic and repetitive reduced voluntary movement and responsiveness to external stimuli, sometimes maintaining subjective awareness. Given the exclusion of organic and other psychiatric disorders causing impaired consciousness, dissociative stupor was diagnosed. She also exhibited involuntary upward eye deviation (a single prolonged episode without recurrence) without an identifiable organic cause, significantly affecting her daily functioning. In the absence of other medical or psychiatric explanations, conversion disorder was diagnosed. In terms of diagnostic systems, the clinical picture was consistent with ICD-10/ICD-11 categories of dissociative amnesia and dissociative stupor, and with DSM-5 categories of dissociative amnesia and functional neurological symptom (conversion) disorder [1, 2, 4].

Initially, the symptoms caused distress, but strong social support facilitated recovery. Given the strong psychological stressors underlying the symptoms, supportive psychotherapy, psychoeducation, and environmental stabilization were considered the most appropriate treatment approach. Pharmacotherapy was not administered in this case, as psychological interventions were given priority. In addition, considering the patient’s age and psychological adaptability, enhancing self-awareness and coping skills was preferred over medication reliance.

As symptoms resolved after *T* =  −3 weeks, supportive psychotherapy without medication was continued, emphasizing psychoeducation and gradual memory reintegration. Outpatient follow-ups focused on supportive psychotherapy, reassurance, and psychoeducation on dissociative symptoms, emphasizing their reversibility. The patient and her caregivers received guidance on coping strategies to manage stress and prevent recurrence. Although she occasionally expressed concerns about memory-related difficulties and future uncertainty, these were addressed through structured discussions and problem-solving strategies.

Her memory steadily improved without recurrence of dissociative stupor or conversion disorder symptoms. Family and teacher reports indicated no significant ongoing stressors beyond her mother’s past suicide attempt and recent depressive exacerbation. As her symptoms improved, she adapted well to daily life at home and school, with only mild residual amnesia. Given her overall progress, additional psychotherapeutic interventions were deemed unnecessary. Outpatient visits continued at her request until full symptom remission. As her memory gradually returned and her daily routine stabilized, the patient expressed a growing sense of relief and regained confidence.

## Discussion and conclusion

This case presents a rare and complex constellation of dissociative symptoms in an adolescent female, comprising generalized retrograde and anterograde dissociative amnesia, dissociative stupor, and conversion disorder. The onset of symptoms was precipitated by significant psychological stress associated with her mother’s depressive relapse and a previously undisclosed traumatic experience—witnessing her mother’s suicide attempt 2 years earlier. The diagnostic process was complicated by generalized amnesia, which obscured the underlying stressor and necessitated careful differential diagnosis to exclude organic and other psychiatric conditions.

In this case, dissociative amnesia extended beyond episodic autobiographical memory, involving semantic and procedural memory impairments, as well as intermittent anterograde memory disturbances. Such widespread memory dysfunction is uncommon and is typically associated with substantial psychological trauma [5, 6].

Prior to the development of amnesia, the patient experienced recurrent episodes of unresponsive states accompanied by a single prolonged involuntary upward deviation of the eyes, which were indicative of dissociative stupor and features of conversion disorder. Although dissociative stupor is rare, it has been documented as a stress-induced condition and may co-occur with other dissociative disorders [7]. The brief, mild desaturation observed during one episode was most plausibly related to shallow breathing during reduced responsiveness rather than intrinsic cardiopulmonary disease. In this case, the patient’s symptoms resolved with supportive psychotherapy and environmental stabilization alone, without the need for pharmacological treatment.

The co-occurrence of dissociative amnesia, stupor, and conversion disorder has been rarely reported in literature. A review revealed few cases with similar symptom combinations, and none closely resembling the multifaceted presentation observed here [5]. This underscores the importance of further clinical investigation into comorbid dissociative disorders, especially in adolescent populations.

The patient’s developmental background was marked by multiple psychosocial stressors, including domestic violence, inconsistent caregiving, and the early assumption of a caregiving role [8]. These factors likely contributed to her susceptibility to dissociative pathology. Psychoeducational interventions aimed at improving emotional regulation and identifying unhealthy relationship patterns were helpful during the recovery process. Continued psychosocial support may play a key role in preventing codependent behaviors and promoting long-term psychological resilience [9].

This case sheds light on important clinical aspects but should be interpreted within the context of several limitations. First, it is a single-case report without long-term outcome data. Second, continuous observation data were not available and emergency evaluation records could not be independently reviewed. Third, formal, standardized rating-scale assessments of depression or other comorbid psychopathology were not conducted, and prolonged video-EEG monitoring was not obtained. Collectively, these constraints limit causal inference and generalizability.

In conclusion, this case highlights the critical role of comprehensive evaluation in atypical manifestations of dissociation. The diagnoses were consistent with ICD-10/ICD-11 categories of dissociative amnesia and dissociative stupor, and with DSM-5 categories of dissociative amnesia and functional neurological symptom (conversion) disorder [1, 2, 4]. Clinicians must thoroughly rule out organic causes and actively investigate psychosocial stressors, even when trauma is initially denied by the patient. A trauma-informed approach and detailed clinical interviews can facilitate accurate diagnosis. Importantly, effective treatment may be achieved without pharmacologic intervention through supportive psychotherapy, psychoeducation, and a stabilizing environment.

## Data Availability

The datasets are available from the corresponding author upon reasonable request.

## Abbreviations

EEG: Electroencephalogram
ICMJE: International Committee of Medical Journal Editors
MRI: Magnetic resonance imaging
